# Hæmorrhoids

**Published:** 1890-03-01

**Authors:** 


### SURGERY.

### HÆMORRHOIDS.

Kossobudski recommends the following ointment for external hæmorrhoids: Chrysaborin 12 gr., Iodoform 5 gr., Extract of Belladonna 9 grs., Vaseline 1 oz. This should be applied several times daily, washing the parts before application with a 2 p. c. carbolic solution. For internal hæmorrhoids he uses suppositories according to the following formula: Chrysaborin 1½ gr., Iodoform ½ gr., Extract of Belladonna 1 gr., Cacao butter xxx grs. It is stated that after two or three months use of these suppositories the majority of cases are cured. Well, we all know that hæmorrhoids are troublesome things, and that they often appear and subside under various remedies, and often when no remedies are used. Possibly the means advocated by Mons. Kossobudski may aid the subsidence of hæmorrhoids, but we doubt whether confirmed piles are ever got rid of without some kind of surgical operation.

